# Endothelial Dysfunction in Chronic Kidney Disease, from Biology to Clinical Outcomes: A 2020 Update

**DOI:** 10.3390/jcm9082359

**Published:** 2020-07-23

**Authors:** Stefanos Roumeliotis, Francesca Mallamaci, Carmine Zoccali

**Affiliations:** 1 Division of Nephrology and Hypertension, 1st Department of Internal Medicine, School of Medicine, AHEPA Hospital, Aristotle University of Thessaloniki, 54636 Thessaloniki, Greece; st_roumeliotis@hotmail.com; 2 CNR-IFC (National Research Council of Italy, Centre of Clinical Physiology, Clinical Epidemiology of Renal Diseases and Hypertension Unit, Reggio Cal., c/o Ospedali Riuniti, 89124 Reggio Cal, Italy; francesca.mallamaci@libero.it

**Keywords:** endothelial dysfunction, nitric oxide, ADMA, SDMA, cardiovascular risk, chronic kidney disease (CKD), end-stage kidney disease (ESKD)

## Abstract

The vascular endothelium is a dynamic, functionally complex organ, modulating multiple biological processes, including vascular tone and permeability, inflammatory responses, thrombosis, and angiogenesis. Endothelial dysfunction is a threat to the integrity of the vascular system, and it is pivotal in the pathogenesis of atherosclerosis and cardiovascular disease. Reduced nitric oxide (NO) bioavailability is a hallmark of chronic kidney disease (CKD), with this disturbance being almost universal in patients who reach the most advanced phase of CKD, end-stage kidney disease (ESKD). Low NO bioavailability in CKD depends on several mechanisms affecting the expression and the activity of endothelial NO synthase (eNOS). Accumulation of endogenous inhibitors of eNOS, inflammation and oxidative stress, advanced glycosylation products (AGEs), bone mineral balance disorders encompassing hyperphosphatemia, high levels of the phosphaturic hormone fibroblast growth factor 23 (FGF23), and low levels of the active form of vitamin D (1,25 vitamin D) and the anti-ageing vasculoprotective factor Klotho all impinge upon NO bioavailability and are critical to endothelial dysfunction in CKD. Wide-ranging multivariate interventions are needed to counter endothelial dysfunction in CKD, an alteration triggering arterial disease and cardiovascular complications in this high-risk population.

## 1. Introduction

Over the last three decades, chronic kidney disease (CKD) has climbed the ranking list of the diseases that are responsible for the burden of mortality of the world population. In 2016, this disease was 13th on the list of causes of death, and in 2040, it is forecasted as the 5th cause of death [1]. With 850 million individuals affected by kidney diseases, CKD is now considered as a major global public priority [2]. Well beyond other risk factors, CKD is per se one of the strongest risk factors for cardiovascular (CV) death, and in 2013, there were 1.2 million CV deaths secondary to CKD [3]. The heavy CV burden in this population is unexplained by traditional risk factors [4]. CKD-specific CV risk factors including sodium retention and volume expansion, low serum albumin, anemia [5], CKD metabolic bone disorder (MBD)-related factors [6], and the accumulation of potentially toxic substances cleared by the kidney [7] all contribute to atherosclerosis, cardiomyopathy, and CV complications in these patients. 

Atherosclerosis is a process that starts at the level of the endothelium [8], which is a dynamic, functionally complex organ involved in the regulation of several important biological mechanisms, including maintenance of vascular tone and permeability, inflammatory responses, immunity, and angiogenesis. Therefore, the preservation of the structure and functions of the endothelial cells is fundamental for vascular health. Endothelial dysfunction (ED), the very basis of atherosclerosis, is evident at an early stage in CKD patients, and the prevalence of this alteration is progressively higher as the disease progresses toward end stage kidney disease (ESKD) [9]. ED worsening across CKD stages is paralleled by an increasingly higher excess risk of CV death in this condition. Indeed, the risk excess for this outcome is 40% (Hazard Ratio-HR = 1.4) in patients with a glomerular filtration rate (GFR) between 45 and 59 mL/min/1.73 m^2^, and 340% (HR = 3.4) in patients with ESKD (GFR < 15 mL/min/1.73 m^2^). Reduced nitric oxide (NO) bioavailability, a gaseous molecule with vasorelaxant, anti-inflammatory, and antithrombotic properties, [10] is a hallmark of ED in CKD. NO plays a fundamental role in ED and arterial remodeling by several mechanisms, including inhibition of platelet aggregation and monocyte adhesion to the endothelial cell, abrogation of low-density lipoprotein (LDL) cholesterol oxidation, and suppression of the hyperplasia and hypertrophy of smooth muscle cells. 

## 2. Basic Molecular Biochemistry of the NO System

**Figure 1:**
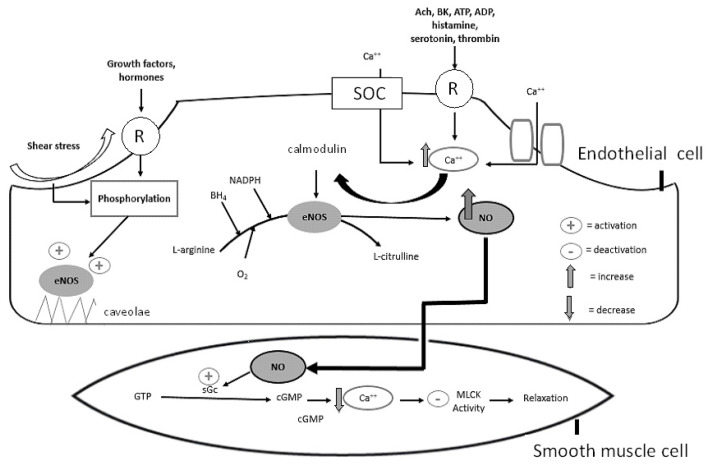
Synthesis of nitric oxide (NO) by endothelial cells. NO is released by the action of endothelial NO synthase (eNOS) on its substrate, L-arginine. For this reaction, several cofactors, such as tetrahydrobiopterin (BH_4_), oxygen (O_2_), and nicotinamide adenine dinucleotide phosphate (NADPH) are required. eNOS is activated by calcium-dependent and -independent pathways. Intracellular Ca is increased as a response to the function of NO agonists (including acetylcholine (Ach), bradykinin (BK), adenosine triphosphate (ATP), and adenosine diphosphate (ADP), serotonin, thrombin, and histamine) or by influx into the endothelial cell following the opening of the store-operated calcium channel (SOC) and other calcium permeable channels in the plasma membrane. Ca binds to calmodulin, and this complex dissociates eNOS from the inhibitor caveolin-1, located in the caveolae. eNOS can also be activated by the calcium-independent pathway of post-translational phosphorylation, which is triggered as a response to shear stress and the function of growth factors and hormones. After NO is generated, it diffuses across the membrane of smooth muscle cells and activates the guanylyl cyclase (sGC), which catalyzes the reaction from guanosine triphosphate (GTP) to cyclic guanosine monophosphate cyclic guanosine monophosphate (cGMP). The effect of this reaction is the transportation of calcium out of the cell, which downregulates the activity of myosin light chain kinase (MLCK), causing relaxation of the vascular smooth muscle cell. Abbreviations: Ach, acetylcholine; ADP, adenosine diphosphate; ATP, adenosine triphosphate; BH_4_, tetrahydrobiopterin; BK, bradykinin; Ca, calcium; cGMP, cyclic guanosine monophosphate; eNOS, endothelial NO synthase; GTP, guanosine triphosphate; MLCK, myosin light chain kinase; NADPH, nicotinamide adenine dinucleotide phosphate; NO, nitric oxide; O2, oxygen; R, receptor; sGC, guanylyl cyclase; SOC, the store-operated calcium channel.

The generation of NO from L-arginine is catalyzed by three NO synthase (NOS) isozymes. Although neuronal NOS (nNOS or NOS-1) is expressed in the nervous system and cytokine-inducible NOS (iNOS or NOS-2) is released by pro-inflammatory cytokines, the primary regulator of endothelium NO bioavailability is the endothelial NO synthase (eNOS or NOS-3). eNOS can be activated by both calcium-dependent and -independent pathways (Figure 1). Several NO agonists including acetylcholine (Ach), bradykinin (BK), adenosine triphosphate (ATP), adenosine diphosphate (ADP), endothelin-1, serotonin, histamine, and thrombin bind to their receptors or open ion channels at sites located on the endothelial cell membrane to increase the influx of calcium ions and stimulate its release from the endoplasmic reticulum, which serves as an intracellular calcium store. eNOS is primarily located in flask-shaped invaginations of the plasma membrane, the caveolae, which contain the caveolin-1 protein. The increase of intracellular calcium re-arranges the conformation of calmodulin (CaM) by forming calcium–calmodulin complexes. The calcium–CaM-dependent dissociation of eNOS from caveolin-1 allows eNOS translocation to the cytosol, where all the cofactors and substrate for activation of this enzyme are in abundance [11]. 

Moreover, the association of this complex with heat shock protein 90 further induces eNOS activity. On the other hand, growth factors (vascular endothelial growth factor (VEGF)), hormones (insulin and adiponectin), and shear stress initiate phosphorylation of eNOS, a post-translational modification that enhances eNOS activity, independently of calcium [12]. Although eNOS is primarily synthesized as a monomer compound, it is necessary to form dimers to bind its substrate and cofactors and achieve NO generation. The dimer consists of two subunits. The first sub-unit is the oxygenase domain with binding locations for zinc, iron protoporphyrin IX (heme Fe), tetrahydrobiopterin (BH_4_), and the substrate L-arginine. The second sub-unit is the reductase domain with binding locations for the cofactors flavin adenine (FAD), flavin mononucleotide (FMN), nicotinamide adenine dinucleotide phosphate (NADPH), and CaM [13]. Molecular oxygen binds to eNOS in the oxidase domain, is reduced by NADPH, and is incorporated in L-arginine to form an intermediate compound, NG-hydroxy-L-arginine, which is finally transformed into L-citrulline, NO, and NADP+. Absence of BH_4_ results in a condition termed eNOS uncoupling, which includes conformation of eNOS from a dimeric to monomer form. In this form, eNOS, instead of NO, releases superoxide anion, a highly reactive free radical. In conditions of low intracellular calcium concentration, the store-operated calcium channel (SOC) is stimulated to absorb extracellular calcium, whereas shear stress activates the calcium/potassium channel to allow calcium influx into the endothelial cell. After its generation, NO diffuses across the membrane of smooth muscle cells and upregulates the expression of the enzyme soluble guanylyl cyclase (sGC), which catalyzes the reaction from guanosine triphosphate (GTP) to cyclic guanosine monophosphate (cGMP). The effect of this reaction is the stimulation of protein kinase G (PKG) that activates transportation of calcium out of the cell and subsequently downregulates the activity of myosin light chain kinase (MLCK), causing relaxation of the vascular smooth muscle cell [10]. However, cGMP-independent signaling, such as S-nitrosylation of proteins; activation of endoplasmic reticulum calcium ATPase; and generation of cyclic inosine monophosphate (cIMP) might also be involved. 

## 3. Major Factors Modulating Bioavailability of NO

ED can be defined as the disturbance of the balance between vasoconstricting and vasodilating molecules generated by or acting on the endothelium. However, since endothelium is a ubiquitous tissue, this disturbance can be regarded as a systemic disorder, characterized by limited NO bioavailability, which could result from either decreased synthesis or increased consumption. Due to the complex mechanism of NO synthesis, several factors downregulate eNOS activity and affect NO bioavailability. 

### 3.1. eNOS, Caveolae, and Ox-LDL

eNOS location in the endothelial cell is crucial for its function. Caveolae are lipid-containing microdomains of the endothelium plasma membrane, where eNOS binds and is directly regulated by the protein caveolin. The activity of eNOS in the caveolae can be regulated by multiple extracellular factors affecting the eNOS–caveolin link. In particular, cholesterol and lipoprotein abnormalities disrupt normal eNOS localization and activity. Oxidized LDL (Ox-LDL), but not native LDL, by binding to cluster of differentiation 36 (CD36) endothelial scavenger cell receptor depletes caveolae of cholesterol, thereby promoting the displacement of eNOS–caveolin from caveolae to the cytoplasm, where eNOS activity is directly inhibited [14,15]. Moreover, in clinical studies, treatment with statins induces a significant reduction in CD36 expression before Ox-LDL changes [16]. In addition to this mechanism, Ox-LDL might also interfere with the production of NO through other cholesterol-independent mechanisms, including downregulation of BH_4_ production, excessive formation of superoxide anions, and disruption of Ach-induced and serotonin-induced activation of eNOS. However, eNOS phosphorylation is not affected by Ox-LDL [17]. In CKD and hemodialysis (HD) patients, Ox-LDL has been inversely associated in an inverse fashion with carotid artery distensibility and smoking in the general population [18]. Similarly, the ox-LDL/apoB100 ratio was found to be inversely related to vascular endothelial function in another population-based study [19], and Ox-LDL and the severity of malnutrition were both independent determinants of flow-mediated vasodilation in HD patients [20]. Moreover, intervention strategies that improve the lipidemic profile (including loss of weight, adaptation of healthy diet, physical activity, and administration of several anti-lipidemic agents) associate improvement of ED through suppression of Ox-LDL levels and oxidative stress (OS) [21,22,23,24,25].

The subcellular redistribution of eNOS, induced by Ox-LDL, might be ameliorated by high-density lipoprotein (HDL) cholesterol through endothelial caveolae scavenger receptor class B, type I (SR-BI)-induced kinase signaling. Moreover, HDL acts as an eNOS agonist through upregulation of post-translational phosphorylation of the enzyme [26]. In the clinical setting, HDL was found to be an independent predictor of endothelial function [27,28]. In contrast, in patients with coronary artery disease (CAD) and HDL depletion, a 25% increase in HDL levels caused by pharmacological treatment with niacin was accompanied by a significant improvement of ED [29]. Moreover, administration of apoA-l/phosphatidylcholine particles in hypercholesterolemic men and ATP-binding cassette transporter in heterozygotes with HDL depletion resulted in significant enhancement of endothelial activity [30,31]. 

### 3.2. Age and Estrogens

Several signal transduction molecules regulating eNOS activity are co-hosted with eNOS within the caveolae, including G protein-mediated and calcium-mediated protein kinase and lipid signaling molecules [32]. Among those, estradiol upregulates eNOS activity via both genomic and non-genomic actions of the caveolin-localized estrogen receptor (ER) alpha, even in the absence of CaM, calcium, and other eNOS cofactors. ER antagonists, antibodies, and calcium chelation [33] inhibit the interaction between eNOS and ER alpha in the caveolae. The mechanisms underlying the beneficial effect of estrogens are probably suppression of OS that increases NO bioavailability and improved responsiveness of smooth muscle cells to vasodilatory stimuli [34,35]. Compared to pre-menopausal women, ED is more pronounced in postmenopausal women with estrogen depletion and age-matched men [36]. The protective effect of estrogens on arterial function might also be due to reduced vascular BH_4_, an essential cofactor for eNOS activation, in postmenopausal women. Supplementation with BH_4_ improved arterial stiffness and increased endothelial-dependent vasodilation of carotid and brachial artery in estrogen-deficient postmenopausal women but did not affect pre-menopausal women [37]. 

### 3.3. Shear Stress

Endothelial cells are under constant mechanical loading from the blood flow (shear stress). Increased endothelial shear stress induced by an increase in blood flow may enhance the activity of eNOS in a calcium-independent way [38,39]. In human coronary circulation, an acute increase of shear stress promotes the generation of BK, which subsequently upregulates the function of eNOS via a Gq-dependent pathway [11,39,40]. Moreover, immediate exposure to shear forces may increase the permeability of human glomerular endothelial cells through upregulation of eNOS activity [41]. However, in animal models, other mechanisms have been suggested to underlie this association, including activation of angiotensin II (AT2) receptors [42], calcium–potassium channels [43], and G protein-coupled receptors [44]. Chronic exposure to increased shear stress enhances eNOS activity through upregulation of the phosphorylation of this enzyme, resulting in higher production of NO. This mechanism might explain the beneficial effects of aerobic exercise training on endothelial function [40,45]. 

### 3.4. BH_4_ and L-Arginine

Depletion of the substrate L-arginine or the essential cofactors for eNOS or increased production and activity of endogenous inhibitors of the enzyme could also limit NO bioavailability. BH_4_ is synthesized from sepiapterin, a reaction catalyzed by the enzyme GTP-cyclohydrolase I (GTPCH I). Prolonged deficiency of circulating BH_4_ has been associated with decreased endothelium-dependent relaxation [37,46]. Likewise, mutation or deletion of GTPCH I leading to BH_4_ depletion decreases NO availability and endothelium-dependent relaxation that could be reversed by sepiapterin administration [47]. BH_4_ deficiency limits NO bioavailability and superoxide anion generation by the uncoupled eNOS. The beneficial effects of BH_4_ administration on endothelial function have been reported in several settings, including post-menopause [37], hypertension [48], CAD [49], dyslipidemia [50], and diabetic vasculopathy [51]. 

L-arginine, the substrate of NO, is a semi-essential amino acid that can be synthesized from L-citrulline, and therefore deficiency of both amino acids may lead to limited bioavailability of NO [52]. In essential hypertension [53], acute supplementation of L-arginine restores the response to Ach, thereby improving NO-dependent vasodilation. Furthermore, L-arginine mitigates platelet aggregation and smooth muscle cell proliferation [54]. However, in patients with peripheral artery disease (PAD), chronic treatment with this amino acid offered no significant therapeutic benefit, and in some cases brought about potential harm [55]. Since experimental data suggest that L-arginine might even trigger senescence of endothelial cells [56], researchers have hypothesized that L-arginine can hardly affect endothelial NO synthesis on a chronic basis. 

### 3.5. Asymmetric Dimethylarginine (ADMA)

ADMA is a potent endogenous inhibitor of eNOS [57]. This dimethylated amino acid is generated in endothelial cells from L-arginine and molecular oxygen, a reaction catalyzed by a family of protein arginine methylation enzymes (PRMTs). Besides competing with L-arginine for eNOS, ADMA also promotes eNOS uncoupling in order to generate free radicals [58]. LDL-cholesterol, OS, and shear stress upregulate the expression of PRMT-1. In contrast, kappa beta inhibitors (κβ-I) kinases (molecules that facilitate the transfer of the κβ-I from the cytoplasm to the endothelial cell nucleus, a location where this factor promotes the release of pro-inflammatory cytokines) have the opposite effect [59]. Besides PRMT-1, plasma ADMA levels are regulated by the enzyme dimethylarginine dimethylaminohydrolase (DDAH), which degrades ADMA into L-citrulline. This reaction is a crucial step in the metabolic pathway of ADMA regulation, and dyslipidemia, impaired glucose control, pro-inflammatory cytokines, reactive oxygen species (ROS), and advanced glycation products (AGEs) all reduce DDAH expression, which eventually results in increased ADMA levels. However, downregulation of DDAH per se does not affect the vascular response to Ach but regulates angiogenesis in endothelial cells [60]. 

### 3.6. Oxidative Stress

Oxygen-derived free radicals are another critical determinant of the bioavailability of NO. Multiple enzymes within the endothelial cells can produce superoxide anions, including xanthine oxidase, NADPH oxidase, and eNOS itself in its uncoupled form, in conditions of L-arginine or BH_4_ deficiency [61]. Moreover, ROS scavenge NO, resulting in the generation of peroxynitrite, which further decreases NO bioavailability and causes oxidative damage in DNA, proteins, lipids, and carbohydrates [62], all alterations that may trigger/amplify inflammation and CV damage. In smokers, diabetics, essential hypertensives, and patients with CAD, the acute administration of vitamin C, a free radical scavenger and an antioxidant, restores the subnormal endothelium-dependent vascular responses to Ach, thus implying that OS plays a pivotal role in this alteration [63,64,65,66]. Moreover, in preclinical and clinical studies, AGEs downregulated eNOS activity through degradation of the mRNA of this enzyme and through direct activation of OS and pro-inflammatory cytokines [67,68]. Moreover, AGE-stimulated production of free radicals could contribute to ED in diabetic ESKD patients by increasing the generation of ADMA [69]. 

### 3.7. Adipokines

The adipose tissue cytokines (adipokines) from fat depots may reach the endothelium via the systemic circulation or move from periadvential fat cells to veins and arteries. Studies in animal models and in vitro have shown that periadvential fat cells synthesize both vasoconstrictor and vasodilatory adipokines [70]. Physiologically these cells release adipocyte-derived relaxing factor (ADRF), a vasodilator acting via endothelium-dependent or endothelium-independent mechanisms, such as vascular smooth cell K channels. Perivascular adipocytes also produce adiponectin, a cytokine that regulates AMP-activated protein kinase and that by this mechanism causes vasodilation and vascular remodeling. Another potentially protective cytokine by fat cells is omentin, a peptide that may suppress NOX (NADPH oxidase) activity and NF (nuclear factor)-κB signaling [71]. On the other hand, perivascular fat cells release interleukin (IL)-6, a pro-inflammatory cytokine that stimulates vascular AT1 (Ang II (angiotensin II)) receptors type 1, thereby triggering medial hypertrophy, while monocyte chemoattractant protein 1 (MCP-1) stimulates neointimal formation [70]. Circulating levels of some these compounds or their concentration in the endothelium have been associated with altered vascular control in descriptive studies in humans. However, their role in vascular function control in human diseases, particularly vascular control by periadvential fat cells [69], remains poorly defined.

### 3.8. cGMP

cGMP, which is generated by the enzyme sGC (see Figure 1), is the main molecular target of NO in the smooth muscle cell [72], and reduced cGMP synthesis in response to NO stimulation may attenuate the vasodilatory response to NO or even generate paradoxical vasoconstriction, such as in the case for the response of the endothelium to acute hypoxia [73]. This paradoxical response may depend on the fact that hypoxia switches sGC from catalyzing the production of cGMP from GTP to being a substrate for inosine to produce 3’,5′-cIMP. Overproduction of cIMP at the expense of cGMP might be responsible for the endothelium-dependent contractions observed in hypoxic conditions.

## 4. Methods of Assessment of Endothelial Function

Direct and indirect methods have been developed to assess ED in clinical studies. The endothelial function may be assessed by functional methods that measure the hemodynamic response of the vascular endothelium to physiological stimuli (such as ischemia) or to the administration of substances (such as Ach or methacholine) that stimulate the generation of NO or by quantifying the circulating levels of molecules that are released by the endothelium into the systemic circulation spontaneously or in response to specific stimuli.

### 4.1. Functional Methods

Flow-mediated vasodilatation (FMD) is based on ultrasound imaging of the brachial artery in two conditions, at baseline and after an acute, temporary arterial occlusion (cuff inflation) in the forearm [74]. This test rests on the observation that there is a direct relationship between the magnitude of the post-occlusion arterial dilation (a physiological response to ischemia dependent on NO generation by the vascular endothelium) [75]. Moreover, FMD in the forearm reflects the endothelial function in the coronary arteries [76], and it is considered a reliable indicator of NO bioavailability in various clinical settings, including CKD, essential hypertension, and CAD [77], with this biomarker having been associated with the CKD stage in patients with renal diseases [9]. Even though it is usefully applied in clinical research for testing NO-dependent vasodilatation, this method has conceptual limitations because of cyclooxygenase [78] and hydrogen peroxide [79], with both influencing FMD by NO-independent mechanisms. Furthermore, the generation of endothelium-induced vasoconstrictors or impaired responsiveness of vascular smooth muscle cells to the NO signal contributes to the magnitude of vasodilation [11]. Therefore, in clinical studies aimed at assessing NO bioavailability experiments, testing of inhibitors of eNOS and cyclooxygenases should be performed [11].

Laser Doppler flowmetry and imaging, a reliable indicator of cutaneous microvasculature function, is based on diffusion and refraction of a laser light beam at a known frequency. By shifting the direction of the laser beam away from the vessels, the change in light wavelength (known as Doppler effects) reflects the amount and velocity of erythrocytes in microvessels. In CKD, abnormal measurements obtained by this method not only detected ED but also predicted CV mortality [80,81]. Although simple and easy to perform, this method provides only an indirect measurement of cutaneous perfusion and not a direct estimation of blood flow. Of note, differences in anatomic structures might cause variability in measurements, leading to a limited reproducibility of the technique [82].

Venous occlusion plethysmography was the first technique to be applied for the evaluation of peripheral endothelial function [83], but it is now rarely used because it is impractical and time-consuming. This method involves inflation of a cuff at both the wrist and the upper arm to block venous drainage. Vessel responsiveness is evaluated by measurement of forearm volume changes in response to reactive hyperemia or infusion of vasoactive compounds [83].

### 4.2. Biomarkers of Endothelial Biology

Markers of OS, inflammation, endothelial–leucocyte cell adhesion molecules, NO levels, inhibitors of eNOS (such as ADMA), and endothelial-specific novel markers are all considered markers of ED. Since there is a well-established, bidirectional association between ED and inflammation, the circulating levels of biomarkers of inflammation parallel the severity of ED. This category includes circulating C-reactive protein (CRP), interleukins (ILs), tumor necrosis factor alpha (TNFα), MCP-1, and CD40 ligand. Perhaps the most endothelial-specific biomarkers of endothelial injury are the cell adhesion molecules, vascular adhesion molecule 1 (VCAM-1), intracellular adhesion molecule 1 (ICAM-1), endothelial selectin, and compounds involved in the coagulation pathway such as plasma fibrinogen and von Willebrand factor (VWF). However, most compounds listed as biomarkers of ED, VWF included, are not specific for the vascular endothelium, and therefore might not reflect ED in the coronary system [84]. 

The most endothelium-specific biomarker is represented by endothelial microparticles (EMPs) [85]. High circulating EMPs reflect increased apoptosis of endothelial cells in atherosclerotic vessels. In patients with cardiac ischemia, circulating EMPs correlate positively with the disease severity and inversely with endothelial function assessed by FMD [86]. Endoglin or CD105 [87,88,89], a receptor for transforming growth factor beta (TGF-β), and Endocan, a soluble proteoglycan secreted by vascular endothelial cells [90], are additional putative biomarkers of ED. Among all biomarkers of ED, ADMA is perhaps the most accepted marker of ED. The issue will be further discussed in other sections of this review.

## 5. The Endothelium in CKD

ED is a systemic process [76] extended to the coronary circulation, peripheral arteries, and the kidney vasculature. The most common form of CKD, nephrosclerosis, is considered to be the renal expression of a systemic disorder of the vascular endothelium [91], with ED being pervasive in patients with CKD [92].

**Figure 2:**
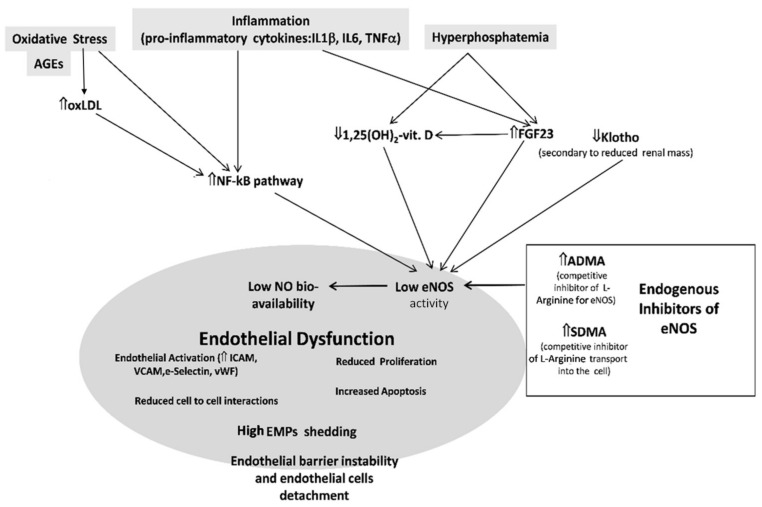
Effect of chronic kidney disease (CKD)-related factors on the vascular endothelium. Oxidative stress and advanced glycation end-products (AGEs), inflammation, low vitamin D, high phosphate and fibroblast growth factor 23 (FGF23), and low Klotho and endogenous inhibitors of eNOS that accumulate in CKD all contribute to reduce NO bioavailability in CKD, thereby triggering endothelial dysfunction (ED). Abbreviations: ADMA, asymmetric dimethylarginine; AGEs, advanced glycation end-products; EMPs, endothelial microparticles; eNOS, endothelial nitric oxide synthase; FGF23, fibroblast growth factor 23; IL-β, interleukin-β; IL6, interleukin6; ICAM, intercellular adhesion molecule; NF-κΒ, nuclear factor kappa-light-chain-enhancer of activated B cell; NO, nitric oxide; Ox-LDL, oxidized low-density lipoprotein; ΤNFα, tumor necrosis factor alpha; VCAM, vascular adhesion molecule; vWF, von Willebrand factor. ⇑ Increase, ⇓ decrease.

Inflammation (a); OS (b); the CKD-MBD including low vitamin D, hyperphosphatemia, high fibroblast growth factor 23 (FGF23), and low Klotho; (c) and the accumulation of endogenous inhibitors on NO synthase, which will be described in a separate section (see below and Figure 2), are the main risk factors underlying this systemic disorder in CKD patients.

In the majority of CKD patients, circulating levels of IL6, CRP, and TNFα are elevated [93,94,95]. As CKD progresses toward kidney failure, the vascular endothelium is gradually activated and releases soluble adhesion molecules—ICAM-1, VCAM-1, and vWF—and matrix metalloproteinases. vWF is increased both in predialysis CKD patients and in HD patients as well [96] and predicts mortality in the dialysis population [97]. Mechanistically, these biomarkers of endothelial activation stimulate the NF-κΒ pathway, which impaired both the endothelial cell lining (cell–cell) and the cell–matrix interrelationships in an in vitro model based on plasma of stage 4–5 CKD patients [98]. Moreover, in the same model, via NF-κΒ-independent pathways, the actin cytoskeleton of endothelial cells was also re-arranged [98]. Inflammation and OS are common in ESKD [99]. Furthermore, at this stage, ED is a universal alteration, which goes along with poor clinical outcomes in the same population [100].

Inflammation in critically ill patients and in severe chronic conditions, including heart failure, CKD, and ESKD, lowers serum triiodothyronine [101,102], and this alteration associates with ED in CKD patients [103]. However, the nature of this relationship remains unclear. In theory, low triiodothyronine may in part mediate the effect of ADMA on endothelial function because the link between triiodothyronine and endothelial function (FMD) in CKD patients was found to be much weaker—but still significant—after statistical adjustment for ADMA levels [103].

AGEs are a heterogeneous group of molecules resulting from OS and glycation processes of proteins, carbohydrates, lipids, and DNA [104]. AGEs bind with a specific receptor, the receptor for AGEs (RAGE). AGEs are raised in CKD patients and correlate inversely with endothelial function, as tested by the response to reactive hyperemia in the skin [105]. Furthermore, skin auto-fluorescence (an estimate of AGE accumulation in the skin) associates with atherosclerosis as quantified by intima-media thickness in non-diabetic individuals without overt cardiovascular disease [106]. Soluble RAGE (sRAGE) detaches from the endothelial surface and circulates free in the blood. These free receptors bind circulating AGEs, thereby exerting a protective, anti-AGE action on the endothelium and on other organs that express the same receptor, such as the heart. Indeed, in CKD patients, RAGE levels are not only inversely related to the severity of atherosclerosis [107], but also to left ventricular mass index [108]. Predictably, AGE levels are very high in ESKD patients, a phenomenon that depends not only on reduced clearance but also on raised endogenous formation attributable to OS and dietary intake of these compounds [109]. Similarly to predialysis CKD patients, ESKD patients display a robust association between accumulation of AGEs in the skin, as quantified by skin auto-fluorescence, as well as pulse wave velocity (PWV) [110]. An important AGE compound such as pentosidine [104] correlates with concentric left ventricular hypertrophy, but not with the severity of carotid atherosclerosis in ESKD patients [111]. This dissociation suggests that this AGE may have direct effects on the myocardium, i.e., effects independent of arterial disease. Furthermore, AGEs have an inhibitory effect on DDAH, the enzyme that degrades ADMA, and the plasma levels of this di-methylated aminoacid are strongly associated with increased AGEs levels in ESKD patients [69].

Vitamin D deficiency is a central alteration in CKD-MBD. The vitamin D receptor is expressed in the vascular endothelium, and vitamin D deficiency in CKD patients has been associated with impaired FMD independently of traditional risk factors [112]. Of note, interventions with active vitamin D compounds improve endothelial function in CKD [113,114]. However, the relevance of these findings for CV prevention in CKD patients remains uncertain because until now there is no clinical trial providing evidence that the use of vitamin D may forestall CV events in this population.

High phosphate levels increase the formation of ROS and suppress the bioavailability of NO through inhibition of eNOS activatory phosphorylation [115]. A small trial in healthy subjects showed that raising serum phosphate by a phosphate-rich meal causes a measurable reduction of flow-mediated vasodilatation [115], and serum phosphate is inversely related to the forearm blood flow response to Ach in essential hypertensives with normal renal function [116]. Likewise, exposure of endothelial cells to high phosphate concentrations triggers the release of EMPs [117]. Of note, a pronounced increase in serum phosphate during paricalcitol treatment abolishes the endothelium-dependent vasodilatation stimulated by this active vitamin D compound in CKD patients [118]. Until now, no trial aimed at reducing serum phosphate showed CV benefits of this intervention in CKD patients, and a clinical trial comparing three phosphate binders raised a safety signal in these patients [119].

FGF23 is a phosphaturic hormone produced by osteocytes and osteoblasts in response to hyperphosphatemia, hyperparathyroidism, hypercalcemia, and inflammation [120]. FGF23 inhibits 1-alpha-hydroxylase, and by this mechanism reduces the synthesis of 1,25 vitamin D [120]. In experimental models, FGF23 hinders NO bioavailability in the endothelium by stimulating the synthesis of superoxide anion [121]. Furthermore, exposure to FGF23—which is a growth factor—triggers hypertrophy in isolated rat myocardiocytes [122]. Accordingly, high circulating levels of this bone hormone correlate with impaired endothelium-dependent vasodilation [123], increased arterial stiffness [124], and left ventricular hypertrophy [125] in CKD patients. However, the implication of high FGF23 for CV diseases remains unclear. Indeed, a systematic review by Marthi et al. [126] failed to show any exposure–response relationship between FGF23 levels and CV events in 17 general population cohorts and an equal number of CKD or dialysis cohorts.

Klotho, an FGF23 cofactor and an anti-ageing protein, is an additional CKD-MBD biomarker that has been implicated in ED and atherosclerosis in CKD and dialysis patients [127]. Klotho-deficient models exhibit reduced NO bioavailability, impaired angiogenesis, and inflammation and accelerated ED [128,129]. Klotho restores NO bioavailability in endothelial cells in vitro and reverts FGF23-induced vasoconstriction in mouse aortic rings [130], suppresses the expression of VCAM-1 and ICAM-1 induced by NF-κB activation [131], and inhibits endothelial apoptosis by increasing the calcium influx into these cells [132]. Furthermore, Klotho mitigates OS in endothelial cells [133]. CKD is a state of Klotho depletion, and low levels of circulating Klotho associate with relatively reduced FMD and thicker carotid intima in healthy subjects [134] and with arterial stiffness [135] and left ventricular hypertrophy in CKD patients [136]. However, whether depletion of Klotho might be an independent risk factor for CV outcomes in CKD remains undefined. Indeed, in a large cohort study in CKD patients by Seiler, FGF23 was a strong predictor of the risk of heart failure while Klotho largely failed to predict CV outcomes in this population [137].

The CKD-MBD in ESKD patients is of almost unique severity. Serum calcium and phosphate, 25OH vitamin D, 1,25 vitamin D, parathormone (PTH), and FGF23 are all profoundly altered in ESKD. London et al. were the first to describe a close association between low 25OH vitamin D, and 1,25 vitamin D and ED in ESKD, with these links being paralleled by equally strong relationships between the same biomarkers and PWV [138]. Of note, hypercalcemia, hyperphosphatemia, high PTH [139], low vitamin levels [140], and high FGF23 [141] have been per se associated with death, as well as CV events, in several studies in ESKD patients. However, FGF23 seems to be the key player in the adverse effects of CKD-MBD in this population because this hormone emerged as a strong risk factor for mortality in analyses adjusting for serum phosphate, PTH, 1,25 vitamin D levels, and treatment with vitamin D compounds [141]. Furthermore, in separate analyses in Caucasians and African American patients, FGF23 coherently emerged as the sole significant CKD-MBD biomarker that predicts death and CV events in the HDs population [142]. However, the precise role of high FGF23 in adverse clinical outcomes in various conditions remains undecided. Indeed, some studies suggest that high FGF-23 may follow, rather than induce, CV disease, as it is the case in myocardial diseases [143].

We remarked that EMPs are the most reliable circulating marker of endothelial damage. In this respect, patients at all CKD stages exhibit increased circulating levels of EMPs [144] and high EMPs reflect defective FMD and increased arterial rigidity as measured by PWV [145]. Endocan, another promising circulating biomarker of ED, has been associated with ED in CKD patients, predicting CV morbidity and mortality more reliably than FMD or carotid intima-media thickness [146]. However, the clinical usefulness of this biomarker remains to be confirmed in other CKD cohorts. Of note, EMPs reflect ED also in ESKD patients [145], potentially being a more reliable marker of endothelial function than other classic markers including NO, eNOS, and endothelin. Such a possibility is suggested by the fact that EMPs in ESKD patients have less production variability and are unaffected by ongoing pharmacologic treatments.

## 6. Endogenous Inhibitors of Nitric Oxide Synthase in CKD

The renal synthesis of the NO precursor L-arginine is reduced in CKD patients [147]. Furthermore, the transport of L-arginine into endothelial cells and the shunting of this amino acid into other pathways, such as those involving arginase, contribute to reducing the availability of this NO precursor (ibidem). In HD patients, mechanical hemolysis occurs, resulting in increased free hemoglobin. Because this oxygen carrier molecule is also an established NO scavenger, free hemoglobin may contribute to reduce NO bioavailability in HD patients [148].

Circulating levels of ADMA and its biologically inactive enantiomer, symmetric dimethylarginine (SDMA), are considerably more increased in CKD, and both compounds are included in the list of “uremic toxins”, i.e., solutes that accumulate in CKD, which can have adverse health effects in the CKD population [149]. ADMA has long been implicated in ED [9], atherosclerosis [150], and CKD progression [151] in this population. Although for a long time research was mainly focused on ADMA, recent studies have shown that SDMA also significantly predicts the risk for CKD and CV disease in CKD patients [152].

As previously alluded to, the primary route of ADMA catabolism is degradation by DDAH, whereas excretion by the renal route is less relevant [153]. In contrast, SDMA is not degraded by DDAH and is primarily removed by renal excretion [153]. SDMA is a robust biomarker of renal function, outperforming the current creatinine-based estimates of glomerular filtration rate (eGFR) (such as CKD epidemiology collaboration (CKD-EPI) and modification of diet in renal disease (MDRD)) and it was equivalent to cystatin C in some studies [154,155]. In a multicenter study in 528 patients with mild to moderate CKD, SDMA outperformed ADMA as a predictor for atherosclerotic CV complications and progression of CKD [152]. The strength of the relationships between SDMA and atherosclerotic events and CKD progression in this study [152] most likely depend on residual confounding by renal function because SDMA is a reliable biomarker of renal function (see above). A meta-analysis based on 17 cohort studies confirmed that SDMA is an independent predictor of mortality and CV disease in various populations, particularly in studies based on the general population [156]. However, in this meta-analysis, SDMA failed to predict mortality and CV events in patients on dialysis, i.e., in a population with minimal or abolished renal function [154]. In contrast, ADMA was a coherent predictor of the same events across various populations [156]. Circulating ADMA is inversely related to flow-mediated vasodilatation in CKD patients [9]. An inverse association between Ach stimulated forearm blood flow and ADMA was also described in patients with essential hypertension and normal renal function [53]. However, the ADMA–forearm blood flow association in CKD patients is wholly independent of arterial pressure [9]. ADMA is not only linked inversely to the eGFR [151] but also associated with CKD complications. In vitro exposure of endothelial cells to erythropoietin (EPO) causes a dose-dependent increase in ADMA levels, and acute administration of EPO to CKD patients triggers an increase in ADMA concentrations [157]. Accumulation of ADMA in erythrocytes in CKD patients might downregulate the activity of EPO receptor, thus leading to lower hemoglobin levels and unresponsiveness to EPO treatment [158]. Interestingly, a recent cross-sectional study in a large cohort of CKD patients suggested that the association between CKD and sleep disorders might be mediated by the accumulation of ADMA in serum [159]. However, the purely cross-sectional design of this study prevents a causal interpretation of the sleep apnea ADMA link.

In animal models of renal disease, a reduction in ADMA levels by manipulation of DDAH translated into better renal outcomes in some models [158,160] but resulted in a paradoxical acceleration of renal damage in other models [161]. Observational studies coherently implicate ADMA in the high risk for the CV and renal outcomes in CKD patients. The previously discussed meta-analysis by Schlesinger [156] gathered 34 studies testing the link between ADMA and mortality and CV events. In this meta-analysis, ADMA predicted death and CV complications in various populations. Furthermore, two studies in 2005 showed that high ADMA predicts a high risk of CKD progression in CKD patients [151,162], and these findings were subsequently confirmed in other studies [152,163]. Interestingly, plasma ADMA and FGF23 are inter-related each other in CKD patients [123,164], and a study based on two CKD cohorts described a competitive interaction between ADMA and FGF23 for the prediction of CKD progression. Both FGF23 and ADMA reduce eNOS activity. Such an interaction suggests that, by unknown mechanisms, when these two factors are elevated they inhibit each other [164], which may be a protective effect aimed at preventing massive NO inhibition and the ensuing organ damage triggered by simultaneously high levels of FGF23 and ADMA.

Although both ADMA and SDMA have similar molecular weight, they are both poorly removed by HD, which depends on the tight binding of these dimethylarginines to plasma proteins [57]. More extended HD has no measurable effect on the removal of these compounds [165], and in this respect, hemodiafiltration is not superior to HD [166,167]. The profile of biomarkers of CV risk differs among HD patients and patients on continuous ambulatory peritoneal dialysis (CAPD). Indeed, plasma ADMA and norepinephrine levels as well as serum CRP levels are all higher in CAPD than in HD patients (reviewed in [168]). The difference in plasma ADMA, a key mediator of NO synthase inhibition in ESKD, among the two populations would in theory entail a 15% higher risk for CV events in CAPD compared to HD patients [168].

## 7. Protecting Endothelial Health in CKD

Given the importance of vascular endothelium as the tissue where atherosclerosis originates and taking into account factors implicated into ED, several compounds have been or are being investigated as endothelium-protective agents.

### 7.1. Lipid-Lowering Drugs

Statins exert a beneficial effect on ED, independently of their cholesterol-lowering effect. Meta-analyses of randomized controlled trials showed that treatment with these drugs reduces circulating biomarkers of ED such as VEGF [169], endothelin-1 [170], and ADMA [171], and that they improve FMD [172]. Moreover, randomized controlled trials in HD patients with eicosapentaenoic acid—an agent considered to increase the stability of LDL cholesterol to peroxidation—showed that treatment with this agent [173] markedly reduces Ox-LDL levels and prevents in vivo peroxidation of LDL in dialysis patients. A trial testing atorvastatin [174] showed the same effect on Ox-LDL levels but not on LDL susceptibility to oxidation. By restoring NO bioavailability, omega-3 polyunsaturated fatty acids might improve ED in CKD [175]. However, all these studies looked at surrogate endpoints rather than at clinical endpoints. There is still no sufficiently large randomized controlled trial examining the effect of omega polyunsaturated fatty acids on clinical outcomes in CKD and ESKD patients. On the other hand, a meta-analysis of the several trials testing statins in CKD and in ESKD patients [176] showed that, in global terms, these drugs cause a 23% risk reduction for major CV events, an 18% risk reduction for coronary events, and a 9% reduction in CV or all-cause deaths. In the same meta-analysis, statins had no significant effect on stroke and no apparent effect on kidney failure events.

### 7.2. Vitamins C and E

Since OS is an essential mediator on the development of ED in CKD, it was hypothesized that supplementation of antioxidants might act protectively on the vascular endothelium. Dialysis patients frequently exhibit depletion of vitamin C, a well-established exogenous antioxidant with anti-inflammatory properties. In a small cohort of children with CKD, administration of vitamin C for 1 month improved carotid intima media thickness (cIMT) and FMD values, thus indicating a favorable effect on ED [177]. Moreover, in HD patients, acute administration of the antioxidant and ROS scavenger vitamin C resulted in improvement of FMD through suppression of OS biomarkers [178]. However, in randomized trials, vitamin C failed to show a clear-cut effect on OS and lipid peroxidation status in HD subjects [179], and no large trial based on clinical endpoints has ever been performed in CKD and ESKD patients.

The Antioxidant Therapy in Chronic Renal Insufficiency (ATIC) study showed that combined supplementation of vitamin E, vitamin B, and pravastatin for 18 months increases FMD and ameliorates carotid atherosclerosis in patients with mild to moderate CKD [180]. Since the exposure of blood to the membrane of dialysis filters contributes to inflammation and OS, researchers have developed vitamin E-coated dialyzers. The use of these filters decreases lipid peroxidation and the pro-inflammatory cytokines CRP and IL6 [181] but has no impact on atherosclerosis as measured by the cIMT [182]. Until now, no study based on clinical endpoints has tested these filters in HD patients.

### 7.3. L-Arginine

In experimental models of obstructive nephropathy and puromycin-induced nephrosis, L-arginine uptake reduces inflammation and increases corticoid release, which are potentially beneficial effects [183]. However, other experimental data suggest that arginine supplementation can worsen tissue injury and subsequent fibrosis in immune-mediated renal disease through a substrate-dependent mechanism [183]. In HD patients, acute L-arginine administration improved Ach-stimulated endothelium-dependent vasodilation [184]. In contrast, L-arginine supplementation failed to improve ED in children [185] and adults with chronic kidney failure [186]. There are no studies that have tested the effect of low protein diets on NO synthesis in CKD patients.

### 7.4. Vitamin D

Activation of the vitamin D receptor by paricalcitol or vitamin D analogues improves ED in a dose-dependent way and reduces left ventricular hypertrophy in the remnant kidney model [187,188,189]. These beneficial effects of paricalcitol in this model are attributed to the fact that in this rat model of CKD-active vitamin D reinforces the intercellular links of endothelial cells, thereby stabilizing the endothelial barrier and preventing endothelial cell detachment [98,190]. The paricalcitol and endothelial function in CKD (PENNY) trial in stage 3-4 CKD patients showed a clear benefit of paricalcitol treatment on FMD [113]. Another randomized trial testing cholecalciferol in vitamin D-deficient CKD patients [191] confirmed the beneficial effect of vitamin D compounds on FMD in this population. However, two randomized trials looking at a robust surrogate marker of CV risk such as the left ventricular mass index—the Paricalcitol Capsule Benefits in Renal Failure–Induced Cardiac Morbidity (PRIMO) [192] and the Oral Paricalcitol Echocardiography Randomized (OPERA) trials—failed to show any effect of paricalcitol on left ventricular hyperthrophy in CKD patients [193]. Likewise, among HD patients without secondary hyperparathyroidism, oral active vitamin D did not offer any significant protection against the occurrence of CV events in the Japan dialysis active vitamin D trial (J-DAVID) [194]. A meta-analysis of studies that tested the effect of various vitamin compounds on FMD in various conditions, CKD included, showed a benefit of these compounds on this outcome measure [195]. However, a recent meta-analysis of 31 trials in various diseases, also considering other biomarkers of arterial function such as PWV, failed to show an improvement in FMD or PWV or other arterial function metrics [196]. Nevertheless, an analysis restricted to the two sole studies testing the effect of paricalcitol on FMD in CKD patients showed a highly significant increase in FMD (*p* = 0.002) by this drug. In the same way as the effect of vitamin D supplementation on human health [197], the effect of the same vitamin on endothelial function and CV disease remains an unsettled question in CKD.

### 7.5. Klotho, FGF23, and Phosphate Binders

In the face of several experimental studies showing the beneficial effects of Klotho in various experimental models [198,199,200,201,202,203], there is no evidence that Klotho has protective effects for the endothelium or the heart and the kidney in CKD patients. Sevelamer, a phosphate binder, reduced serum phosphate and increased FMD in hyperphosphatemic CKD stage 4 patients [204]. However, whether mitigating hypophosphatemia may translate into better CV outcomes in CKD patients remains unknown. The issue is of crucial importance because a previously discussed trial comparing four phosphate binders in predialysis CKD patients [119], along with a mild reduction in serum phosphate, showed a rise in vascular calcification in patients treated with these drugs as compared to those treated with placebo.

### 7.6. Antihypertensive and Antidiabetic Agents

Several categories of antihypertensive drugs, including calcium channel blockers [205], angiotensin-converting enzyme (ACE) inhibitors, or angiotensin II receptor blockers [206,207,208] and ultra-selective β1-blockers such as nebivolol [209], have been shown to reverse ED in essential hypertensives. Calcium channel blockers enhance the expression of NO synthase in endothelial cells [210], and drugs interfering with the renin–angiotensin system improve ED by multiple mechanisms including antagonism of the direct vasoconstrictive effect of angiotensin II; the reduction of endothelin I synthesis, another potent vasoconstrictor; and the attenuation of angiotensin II-triggered NF-κΒ signaling and OS. The effects of these drugs on the endothelium are of utmost clinical relevance. Indeed, two meta-analyses, one focusing on calcium channel blockers [211] and one focusing on ACE inhibitors and angiotensin II blockers [212], documented the fact that these drugs prevent atherosclerosis progression. The effect of ACE inhibition and angiotensin II receptor blockers on the endothelial function has been very minimally studied in CKD and ESKD patients. Small studies in predialysis CKD patients with type-2 diabetes [213] and ESKD patients on HD [214] produced conflicting results because ACE inhibition by ramipril improved FMD and reduced ADMA levels in the first study [213] whereas it increased ADMA in the second [214]. In contrast, the angiotensin II blocker valsartan did not modify ADMA in the same study [214]. Nebivolol ameliorates ED through several pathways, including suppression of OS and ADMA and upregulation of eNOS expression [215,216,217,218]. No studies on the effect of nebivolol on endothelial function or CV outcomes have been performed in CKD patients.

Newer antidiabetic agents, such as sodium-glucose cotransporter-2 (SGLT-2) inhibitors [219,220] and glucagon-like peptide-1 receptor agonists (GLP-1 RAs) [221], decreased PWV in studies in patients with type-2 diabetes. Saxagliptin, a dipeptidyl peptidase-4 (DPP-4) inhibitor, prevented vascular remodeling and OS in a genetic model of diabetes [221].

### 7.7. Renal Transplantation

At least three longitudinal studies documented a clear improvement in endothelial function as measured by the FMD in response to ischemia in HD patients. As previously stated, ED is of almost unique severity in ESKD patients, with dialysis treatments largely failing to meaningfully improve this alteration. Renal transplantation is the sole treatment that can at least partially reverse ED in this population [222,223,224].

### 7.8. Novel Therapeutic Approaches

Activators of soluble guanyl cyclase—the intracellular receptor of NO—improve endothelial function independently of NO because these drugs directly enhance cGMP production [225]. A drug of this class, vericiguat, reduced death from CV causes or hospitalization for heart failure in a large trial in patients with heart failure [226]. Finally, both in uremic animals and humans, microRNAs have emerged as significant mediators of uremia-induced ED [227,228,229], thereby constituting a potential therapeutic target to ameliorate endothelial injury and arterial disease in CKD.

## 8. Conclusions

The vascular endothelium is a crucial defense system against atherosclerosis. In CKD, damage of the vascular endothelium occurs early, develops along with the progression of the disease, and substantially contributes to CV complications in these patients. ED in CKD patients reflects multifactorial endothelial injury coupled to impaired endothelial repair and regeneration. The pathways underlying these processes involve various uremic toxins such as endogenous inhibitors of eNOS, pro-inflammatory cytokines and OS, AGEs, phosphate and FGF23, and subnormal levels of factors protecting the vascular endothelium like Klotho and vitamin D.

A wide-ranging multivariate intervention, including suppression of OS and inflammation, correction of mineral homeostasis disturbances, and removal of toxins, would be necessary to effectively counter or prevent ED and the resulting atherosclerotic complications in CKD. However, limited progress has been achieved over the last 30 years for the prevention of atherosclerotic and non-atherosclerotic CV complications in this population [230], and the issue remains a public health priority [231].
